# Plough Fracture (Anterior Arch Fracture Type Traumatic Posterior Atlantoaxial Dislocation, TPAD‐AOT Type III): A Retrospective Study of Nine Clinical Cases With Complete Imaging Data

**DOI:** 10.1155/bmri/5666198

**Published:** 2026-04-24

**Authors:** Tingfei Yan, Xianyong Wu, Yun Wang, Haisong Yang

**Affiliations:** ^1^ Department of Orthopedics, 411 Hospital, Shanghai University, Shanghai, China, shu.edu.cn; ^2^ Department of Orthopedics, The First Affiliated Hospital of Wannan Medical College, Yijishan Hospital, Wuhu, China, wnmc.edu.cn; ^3^ Department of Radiology, Second Affiliated Hospital of Navy Medical University, Shanghai, China

**Keywords:** hyperflexion-hyperextension injury, plough fracture, therapeutic management, transverse ligament injury

## Abstract

Fracture of the anterior arch of the atlas, accompanied by posterior atlantoaxial dislocation, typically presents on CT imaging as the odontoid process penetrating through and causing a high‐energy shear fracture of the anterior arch. This injury may occur with or without retained fracture fragments. Its characteristic appearance resembles that of a plough traversing the earth, and it has historically been termed the plough fracture. Published case reports documenting this specific fracture pattern remain extremely rare in the literature. In our research group′s earlier study on traumatic posterior atlantoaxial dislocation (TPAD), this fracture pattern was categorized as anterior arch fracture type TPAD (TPAD‐AOT Type III**)**. It was further subclassified based on transverse ligament integrity into: (1) Anterior arch fracture type TPAD with intact transverse ligament, and (2) Anterior arch fracture type TPAD with transverse ligament injury, reflecting a progressive increase in instability severity across these subtypes. Hyperflexion and hyperextension injury mechanisms play significant roles in the pathogenesis of this fracture pattern, with definitive diagnosis typically established via CT imaging. The integrity of the transverse ligament is critical for atlantoaxial stability. For patients with an intact transverse ligament, rigid external fixation or internal fixation may be indicated based on fracture displacement and reducibility. However, in cases with transverse ligament injury, posterior C1–C2 fusion typically achieves favourable functional outcomes.

## 1. Introduction

Traumatic posterior atlantoaxial dislocation with fracture (TPAD with fracture) represents an exceptionally rare clinical entity encountered in emergency departments. This condition was first described by Fielding and Hawkins et al. as occurring when severe trauma compromises the odontoid process or anterior arch of C1, resulting in sagittal plane instability and posterior displacement of C1 relative to C2 [1]. Traumatic posterior atlantoaxial dislocation (TPAD) is commonly associated with fractures of the odontoid process and/or the anterior arch of the atlas. Current clinical literature predominantly reports cases involving odontoid fractures, whereas anterior arch fractures remain relatively uncommon, with only a limited number of published cases [2–4].

Fractures of the anterior arch of the atlas accompanied by posterior atlantoaxial dislocation typically manifest on CT imaging as the odontoid process transfixing the arch, resulting in a high‐energy shear fracture. This injury pattern may occur with or without retained fracture fragments, and its characteristic appearance resembling a plough traversing soil has led to its designation as the “Plough fracture” [3, 4]. Mohit et al. first described a case of atlas Plough fracture with concomitant comminuted posterior arch fracture in 2003 [3]. In contrast, the inaugural report of an isolated anterior arch plough fracture (without posterior arch or odontoid process involvement) was published by Jason et al. in 2004 [5]. To date, only three such isolated cases have been documented in the literature [2, 5, 6].

In our research group′s prior investigations on TPAD, this injury pattern was classified as anterior arch fracture type TPAD (TPAD‐AOT Type III) [7]. In this injury pattern, free‐floating anterior arch fracture fragments compromise the containment of the odontoid process, predisposing to anterior displacement and resultant atlantoaxial instability. This instability significantly elevates the risk of potentially life‐threatening cervical spinal cord and vertebral artery injuries. The severity of these injuries contributes to substantial prehospital mortality, preventing many patients from reaching medical facilities. Consequently, the true incidence of plough fractures is likely significantly higher than currently documented in the literature. The aim of this study is to conduct a systematic review of 15 consecutive plough fracture cases from three spine centres over the past decade, analysing the clinical presentations, injury mechanisms, demographic characteristics, diagnostic approaches, management strategies, treatment outcomes and long‐term prognoses associated with this entity.

## 2. Materials and Methods

**Figure 1:**
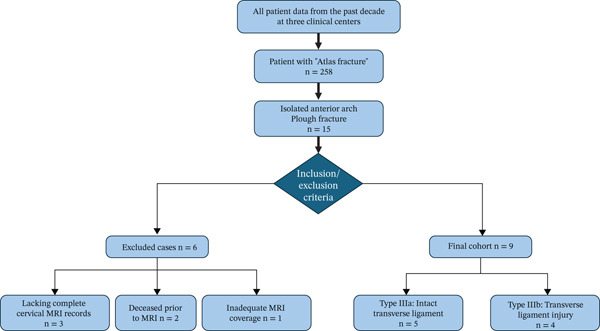
Flow diagram.

In a 10‐year retrospective cohort study, inclusion criteria required anterior arch fractures of the atlas with concomitant posterior atlantoaxial dislocation, whereas exclusion criteria comprised combined posterior arch fractures, associated odontoid process fractures or incomplete radiographic documentation (x‐ray, cervical CT and MRI). Among 258 patients with atlas fractures, CT imaging identified 15 cases of isolated anterior arch plough fractures without posterior arch or odontoid involvement. Subsequent exclusions included three patients lacking cervical MRI records, two who succumbed to critical conditions before MRI completion and one with inadequate craniocervical junction coverage on MRI sequences, resulting in nine patients with complete imaging datasets ultimately qualifying for analysis (Figure 1). We assessed injury mechanisms, imaging characteristics and clinical pathologies in the cohort, with specific evaluation of primary lesions accompanied by ligamentous or neurological injuries. Additionally, treatment modalities and clinical outcomes were analyzed. Finally, a systematic review was conducted of English‐language case reports and series documenting anterior arch fractures of the atlas with concomitant posterior atlantoaxial dislocation in adult patients.

## 3. Results

Trauma imaging and surgical documentation, along with follow‐up imaging and electronic health records, were analyzed for nine consecutive patients with anterior arch fractures of the atlas combined with TPAD. Patients ranged in age from 50 to 69 years, with six males and three females (Table 1). Based on the integrity of the anterior arch‐odontoid‐transverse ligament complex, TPAD injuries were classified into two distinct ligamentous injury patterns, reflecting a progressive increase in instability severity [7] (Table 2).

**Table 1:** Summary of clinical data from published cases of atlas plough fractures.

	Sex	Age	Injury mechanism	Trauma characteristics	Neurological deficit	Comorbid injuries	Imaging	Displacement grade	Transverse ligament	Treatment	Outcome
Jason 2004	F	53	Motor vehicle accident	Forehead impact	Neck pain	None	CT	Grade IV	Not specified	Posterior C1–C2 Magerl transarticular screw fixation with modified Brooks fusion	Full recovery (3 months)
Sasaka 2006	M	40	Motor vehicle accident	Vertex impact	None	Left occipital condyle fracture	XR/CT	Grade II	Intact	Miami‐J collar	Full recovery (4 months)
Ghailane 2019	M	89	Stair fall	Not specified	Neck pain	Not specified	XR/CT	Grade IV	Intact	Manual reduction + cervical collar	Full recovery (8 months)

**Table 2:** Clinical details of nine plough fracture patients in current study.

	Sex	Age	Injury mechanism	Trauma characteristics	Neurological deficit	Comorbid injuries	Imaging	Displacement grade	Transverse ligament	Treatment	Outcome
Case 1	M	63	Motor vehicle accident	Fronto‐parietal hematoma	Incomplete paralysis	Thoracic fracture	XR/CT/MRI	Grade III	Intact	Atlantoaxial fixation	Full recovery (30 months)
Case 2	F	57	Bicycle fall	Craniofacial laceration	Incomplete paralysis	Pleural effusion	XR/CT/MRI^a^	Grade I	Intact	Atlantoaxial fixation	Full recovery (12 months)
Case 3	M	58	Motor vehicle accident	Scalp laceration	Mild motor weakness	Spinal/rib fractures	XR/CT/MRI	Grade III	Intact	Atlantoaxial fixation	Full recovery (18 months)
Case 4	M	61	Bicycle fall	Facial abrasions	Neck pain	None	XR/CT/MRI	Grade III	Intact	Rigid collar	Neck pain(25 months)
Case 5	M	53	Bicycle fall	No visible trauma	None	Ribs/thoracic spine	XR/CT/MRI	Grade II	Intact	Manual reduction + collar	Lost to follow‐up
Case 6	F	50	Bicycle fall	No visible trauma	None	Occipital condyle fracture	XR/CT/MRI	Grade I	Injury (avulsion fracture)	Atlantoaxial fusion	Full recovery (6 months)
Case 7	M	69	Motor vehicle accident	Facial abrasions + frontal hematoma	None	Frontal lobe contusion	XR/CT/MRI	Grade I	Injury (mid‐substance)	Cervical collar	Full recovery (13 months)
Case 8	F	68	Bicycle fall	Craniofacial laceration	Mild motor weakness	Metacarpal/ulnar fractures	XR/CT/MRI	Grade I	Injury (mid‐substance)	Cervical collar (poor surgical tolerance)	Death of pulmonary infection
Case 9	M	59	Motor vehicle accident	Facial abrasions	Incomplete paralysis	C5 Facet Fracture	XR/CT/MRI	Grade IV	Injury (avulsion fracture)	Atlantoaxial fusion	Full recovery (6 years)

^a^MRI images unavailable; complete diagnostic records preserved in clinical documentation.

### 3.1. Anterior Arch Fracture of the Atlas With Posterior Atlantoaxial Dislocation, Without Transverse Ligament Injury (Anterior Arch Fracture TPAD, TPAD‐AOT Type IIIa)

**Figure 2:**
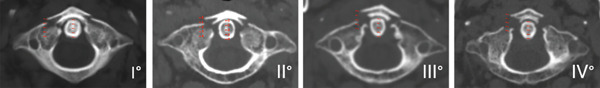
Diagram of displacement magnitude in posterior atlantoaxial dislocation. Based on the displacement distance of the atlas relative to the anteroposterior diameter of the odontoid process, four grades are defined: Grade I: subluxation ≤ 25% of the odontoid anteroposterior diameter; Grade II: subluxation > 25% but ≤ 50%; Grade III: subluxation > 50% but ≤ 75%; and Grade IV: subluxation > 75%.

**Figure 3:**
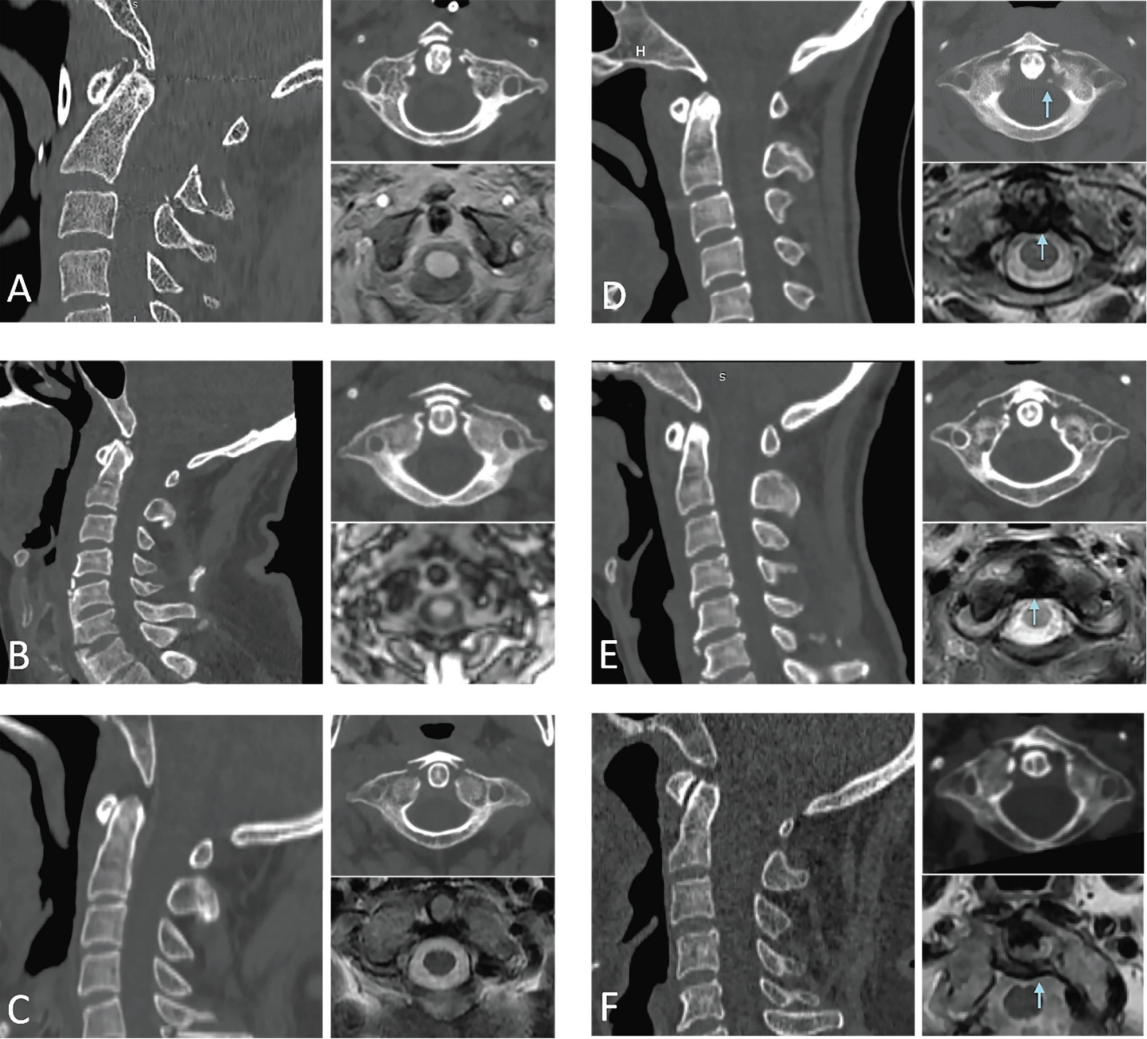
Representative imaging studies of patients 3–8. (A–C) Anterior arch fracture with posterior atlantoaxial dislocation, without transverse ligament injury (TPAD‐AOT Type IIIa). (D–F) Anterior arch fracture with posterior atlantoaxial dislocation, with transverse ligament injury (TPAD‐AOT Type IIIb).

**Figure 4:**
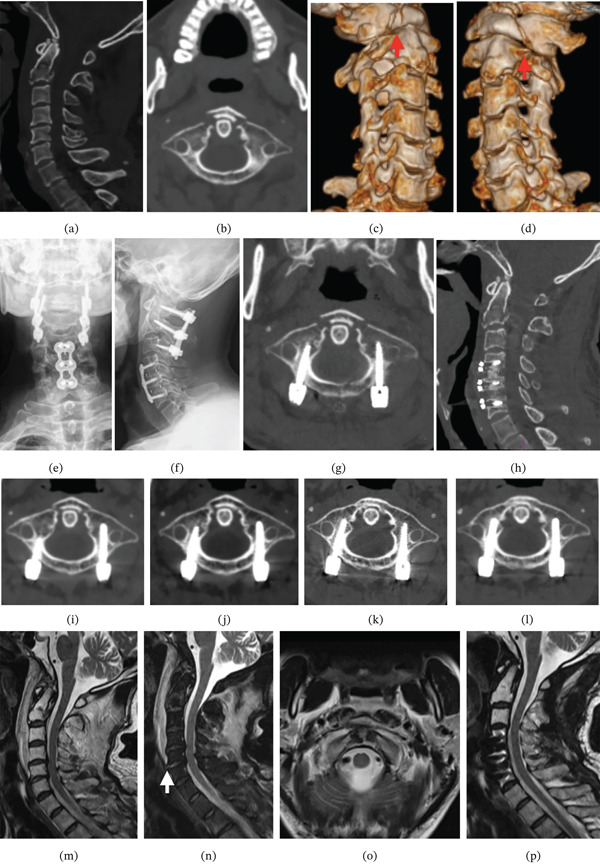
Case example (intact transverse ligament): (a–b) CT sagittal reconstructions demonstrate normal atlantodental interval, reduced basion‐odontoid distance and concurrent posterior dislocation of atlas/occiput; axial views show anterior arch fracture at the arch‐lateral mass junction with odontoid displacing anteriorly carrying an avulsed fragment. (c–d) 3D reconstructions reveal bilateral anterior arch fractures with displaced fragments (red arrows). (e–f) Postoperative radiographs: open‐mouth view shows symmetric atlantodental joints, lateral view confirms no cervical instability. (g–h) Postreduction CT: axial confirms anatomic anterior arch reduction, sagittal demonstrates restored alignment. (i–l) Serial axial CT (6/18/24/30 M) documents progressive anterior arch union with maintained normal ADI. (m–o) Preoperative cervical MRI: posterior cord displacement, hyperintense posterior atlantoaxial soft tissues, capsular ligament injury, intact transverse ligament without disruption, retropharyngeal edema. (p) Postoperative MRI: normal cord morphology without compression.

Five patients (four males, one female; age range 53–63 years) sustained this injury pattern through high‐energy mechanisms (all motor vehicle collisions). Characteristic facial and frontal soft tissue injuries were consistently observed. CT imaging demonstrated bilateral fractures near the anterior margin of the odontoid process with posterior C1–C2 dislocation (Figure 2). Displacement grading revealed: Grade I spondylolisthesis (*n* = 1), Grade II (*n* = 1) and Grade III (*n* = 3) (Figures 3A–C and 4). MRI confirmed intact transverse ligaments in all cases. Three patients underwent atlantoaxial internal fixation. The remaining two achieved successful closed reduction followed by 3‐month halo‐vest immobilization, with one lost to long‐term follow‐up.

### 3.2. Anterior Arch Fracture of the Atlas With Posterior Atlantoaxial Dislocation, With Transverse Ligament Injury (Anterior Arch Fracture TPAD, TPAD‐AOT Type IIIb)

**Figure 5:**
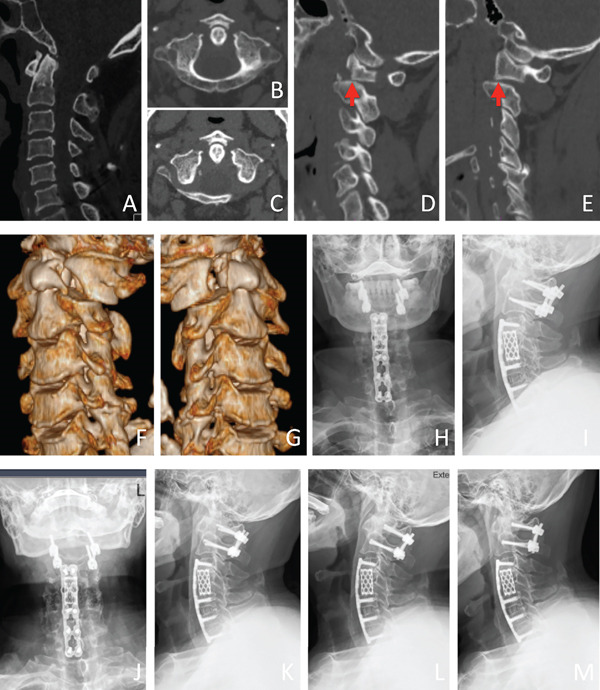
Case Example (transverse ligament injury): (A) CT sagittal reconstruction shows normal atlantodental interval, reduced basion‐dontoid distance and concurrent posterior dislocation of atlas/occiput. (B–C) Axial CT reveals anterior arch fracture at the arch‐lateral mass junction with odontoid displacing anteriorly carrying an avulsed fragment, plus transverse ligament insertion avulsion fracture (arrows). (D–G) Sagittal/3D reconstructions demonstrate bilateral anterior arch fractures with posterior C1–C2 displacement (red arrows). (H–I) One‐year postoperative radiographs confirm proper occipitocervical alignment and hardware position. (J–M) Four‐year follow‐up radiographs (AP/lateral/dynamic flexion‐extension views) show intact instrumentation without loosening/fracture and solid fusion mass formation.

Four patients (two males, two females; age range 50–69 years) sustained similar high‐energy trauma with frequent craniofacial soft tissue injuries. CT showed consistent bilateral odontoid‐margin fractures and C1–C2 posterior dislocation (Figure 1). Displacement grading: Grade I (*n* = 3) and Grade IV (*n* = 1). MRI confirmed transverse ligament injuries in all cases: two demonstrated avulsion fractures at ligamentous insertions on CT, with heterogeneous signal alterations in the tectorial membrane on MRI (Figures 3D–F and 5). Two patients with incomplete paralysis underwent atlantoaxial arthrodesis, maintaining neurological integrity at 1‐year follow‐up. Two neurologically intact patients with poor surgical tolerance received postreduction halo‐vest immobilization for 3 months, with one lost to long‐term follow‐up.

## 4. Discussion

Anterior arch fractures of the atlas are primarily classified into two patterns: vertical fractures (Jefferson fractures) induced by axial loading forces and horizontal fractures resulting from hyperextension mechanisms [8–13]. All nine cases in this series exhibited the rare combination of anterior arch fractures with intact posterior arches and lateral masses, accompanied by posterior atlantoaxial dislocation and functional craniocervical pseudosubluxation—a biomechanical equivalent to occipitocervical dissociation rather than true atlanto‐occipital dislocation. This injury pattern defies conventional classification systems. Radiographically, the odontoid process was observed transfixing the anterior arch, inducing a high‐energy shear fracture with or without retained fracture fragments. The characteristic appearance resembling a plough traversing soil corresponds to the historically described plough fracture [3, 4].

Plough fractures with an intact odontoid process and preserved posterior arch represent an exceptionally rare variant, with only three documented cases in the current literature. In our series, this fracture pattern constituted approximately 5.8% (15/258) of all atlas fractures. We posit that even when the transverse ligament remains intact, the failure of free‐floating anterior arch fragments to constrain anterior displacement of the odontoid process predisposes to atlantoaxial instability. This biomechanical compromise significantly elevates the risk of life‐threatening cervical spinal cord and vertebral artery injuries, resulting in substantial prehospital mortality that prevents most patients from reaching medical facilities—thereby contributing to significant underreporting of this injury pattern [14]. Compounded by the current lack of standardized classification systems for atlas fractures—which contributes to diagnostic ambiguity and frequent underdiagnosis of this injury pattern—the true incidence of plough fractures is likely substantially underreported in the literature. Notably, two fatalities occurred among the excluded cases in our cohort due to severe neurological compromise following hospital admission, further underscoring the life‐threatening nature of this injury.

### 4.1. Injury Mechanism and Fracture Typology

Plough fracture patients typically present with facial and frontal soft tissue injuries, indicating impact forces directed anteriorly rather than vertex loading. This injury profile fundamentally differs from the axial compression mechanism characteristic of Jefferson fractures. Plough fracture patients typically present with facial and frontal soft tissue injuries, indicating direct anterior impact and resultant cervical hyperextension rather than the axial compression characteristic of Jefferson fractures [8]. The fundamental mechanism involves the odontoid process maintaining anterior inertia against the posteriorly translating atlas, generating high shear forces. In our cohort, all nine cases resulted from vehicular trauma, with advanced patient age potentially serving as a contributing factor. Osteoporosis in elderly patients may predispose to craniovertebral junction fractures by facilitating shear forces generated by the odontoid process against the anterior arch.

**Figure 6:**
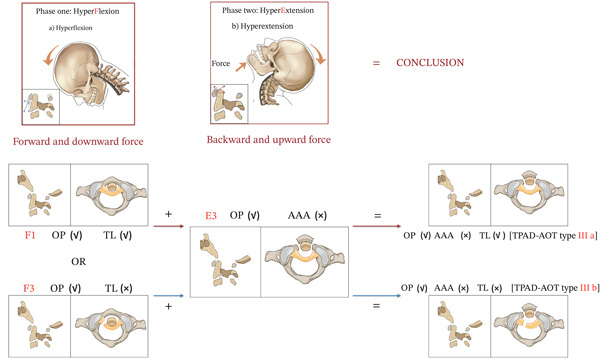
Injury mechanism for plough fracture (anterior arch fracture TPAD, TPAD‐AOT Type III). Stage 1: Cervical hyperflexion forces induce anterior translation of the atlas, causing the odontoid process to exert a guillotine‐like shearing effect on the transverse ligament. Biomechanical compromise of the ligament under sustained hyperflexion may precipitate ligamentous injury. Stage 2: Subsequent hyperextension forces generate posteriorly directed shear stress on the anterior arch, resulting in anterior arch fractures with persistent posterior atlas displacement—manifesting as fracture‐type TPAD. Stage 3: The synergistic action of Stage 1 hyperflexion and Stage 2 hyperextension mechanisms produces the pathognomonic plough fracture of the anterior arch, with or without transverse ligament injury.

**Figure 7:**
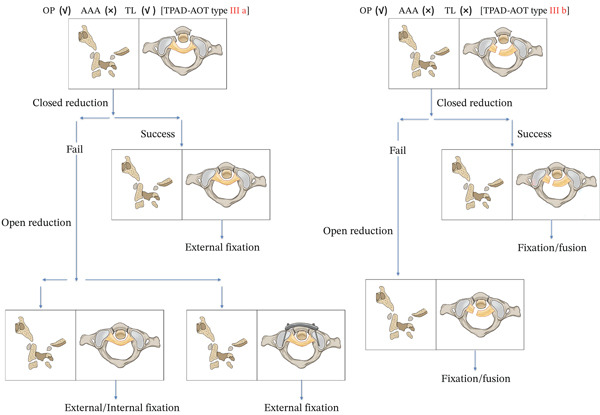
Therapeutic principles for plough fracture (anterior arch fracture TPAD, TPAD‐AOT Type III).

Our research group posits that hyperflexion and hyperextension injury mechanisms play pivotal roles in the pathogenesis of this fracture pattern. Classified as anterior arch fracture type TPAD (TPAD‐AOT Type III), plough fractures fundamentally represent a variant of TPAD. Rather than isolating its injury mechanism, this study synthesizes it within the unified biomechanical framework established for other TPAD subtypes, as previously elucidated in our prior investigations [7]. Taking vehicular trauma as an exemplar, the injury sequence is summarized as follows: Stage 1—As the craniocervical complex sustains hyperflexion forces, anterior translation of the atlas enables the odontoid process to exert a guillotine‐like shearing effect on the transverse ligament. When biomechanically compromised, the transverse ligament becomes susceptible to disruption under persistent hyperflexion loading. Stage 2—Under hyperextension forces applied to the cervical spine, posteriorly directed shear forces acting horizontally on the anterior arch may induce anterior arch fractures. This results in posterior displacement of the atlas, manifesting as fracture‐type TPAD [15]. Patients with this injury pattern frequently present with facial trauma, particularly involving the lower facial region and chin, indicating direct anterior impact that induces cervical hyperextension (biomechanical mechanism detailed in Figures 6 and 7). In all nine patients of this series, the posteriorly displaced Wackenheim line with preserved occipitoatlantal alignment (functional craniocervical pseudosubluxation) further supports this anteroposterior shear mechanism over pure compression.

This mechanistic understanding underscores why the integrity of the transverse ligament is the pivotal biomechanical determinant of stability. Current consensus holds that transverse ligament integrity serves as the crucial biomechanical determinant for stability assessment in atlas fractures: Lesions with intact ligaments are classified as stable fractures, whereas those with ligamentous disruption represent unstable injuries [16].

### 4.2. Clinical Presentation and Diagnosis

Patients in this series and published reports predominantly presented with occipital‐cervical pain accompanied by restricted craniocervical mobility, frequently demonstrating pathognomonic craniofacial or cephalic contusions. A subset exhibited posttraumatic coma (3/9 cases), with some sustaining concomitant lower cervical hyperflexion‐hyperextension injuries. These fractures uniformly resulted from high‐energy trauma mechanisms, with most patients sustaining multisystem injuries (5/9 cases) such as rib fractures and thoracolumbar spinal injuries. CT with multiplanar reconstruction and 3‐dimensional reconstruction is always the gold standard in identifying the bony structures and provides excellent visualization of the rare dislocation. Consequently, in geriatric patients sustaining both high‐energy and low‐energy trauma, posttraumatic CT imaging appears warranted to exclude occult fractures. Although atlantoaxial dislocation patterns may obscure comprehensive assessment of all injured structures, MRI remains indispensable for definitive evaluation of spinal cord integrity and associated soft tissue injuries. Furthermore, MRI findings may provide critical evidence of transverse ligament injury, which provides particularly critical guidance for subsequent treatment selection [17, 18].

### 4.3. Treatment

Theoretical principles mandate prompt fracture reduction in all cases to prevent residual instability and permanent deformity. This study identifies fracture displacement magnitude and transverse ligament integrity as pivotal determinants for treatment selection. In patients with intact transverse ligaments and minimal fracture displacement, conservative management remains the primary approach, with rigid external immobilization typically achieving satisfactory outcomes. However, given the inherent biomechanical instability of this injury pattern, optimal surgical timing constitutes a critical focus in our therapeutic algorithm.

Although anterior transoral reduction and fixation may address plough fractures, this approach carries substantial technical challenges and infection risks. Transoral anterior reduction and fixation can be used to treat teardrop fractures—and in ideal circumstances, it achieves fracture reduction with rigid fixation while preserving cervical rotation—this approach entails significant technical challenges and infection risks. Firstly, the anterior procedure requires a transoral approach. The resident bacteria in the oral cavity substantially increase the risk of infection, whereas the limited thickness of the retropharyngeal soft tissues cannot accommodate bulky internal fixation devices; consequently, inadequate fixation may lead to implant failure. Secondly, the deep and narrow operative field within the oral cavity places extremely high demands on the surgeon′s experience and skill, further increasing the procedural difficulty. Thirdly, because the procedure involves incising the posterior pharyngeal wall, patients require postoperative nasogastric tube feeding, which significantly increases discomfort and raises the risks of aspiration and reflux. Given that anterior arch fracture‐dislocations disrupt the sagittal stability of the occipitoatlantoaxial complex—functionally equivalent to osseous transverse ligament failure—restoration of longitudinal cervical alignment requires reduction of anteroposterior displacement. Consequently, atlantoaxial arthrodesis represents the preferred stabilization strategy. Hirose et al. reported surgical management of a plough fracture patient with neurological deficits. Preoperative evaluation confirmed intact occipitoatlantal articulation and absence of axis fractures. Following 1 week of cranial traction, posterior C1–C2 reduction and fusion was performed. Three‐month postoperative CT demonstrated solid atlantoaxial fusion with documented neurological improvement [4]. Given the life‐threatening potential of this injury pattern, surviving cases likely possess partially preserved ligamentous structures—particularly an intact transverse ligament—that prevent further posterior translation of the odontoid process, thereby avoiding critical narrowing of the spinal canal and neural compromise. When transverse ligament injury exists, ligamentous healing remains challenging even after osseous union through conservative management [19–21]. Among 22 reported cases undergoing transoral miniplate fixation for atlas fractures, solid fusion was universally achieved. However, three patients with transverse ligament disruptions developed atlantoaxial dislocation within 3 months postoperatively. Thus, it is advocated that transverse ligament rupture or bony avulsion at its insertion sites constitutes a definitive indication necessitating surgical arthrodesis [14, 22]. In our research group′s prior cohort of patients with complete posterior atlantoaxial dislocation without odontoid fractures, clinical observation similarly revealed that individuals sustaining concomitant transverse ligament injuries exhibited heightened risk of recurrent atlantoaxial dislocation following anatomical reduction [7]. Consequently, our team endorses the necessity of surgical arthrodesis when transverse ligament injury is documented. In two patients with concomitant transverse ligament injuries, profound atlantoaxial instability necessitated C1‐C2 instrumented fusion. Postoperative CT demonstrated favourable alignment of the occipitoatlantoaxial complex. Long‐term surveillance revealed maintained implant position, osseous union of the anterior arch fracture and full resumption of occupational and daily activities without pain‐related limitations. As shown in the present cohort, surgical management appears to come along with good outcome.

## 5. Limitations

The current paucity of robust investigations into this injury pattern has resulted in limited understanding of its mechanistic underpinnings and optimal management strategies. The primary limitations of our study stem from its retrospective design and small cohort size. Given these constraints coupled with the nascent understanding of this entity, our experience remains preliminary. Consequently, this investigation precludes comprehensive discussion regarding specific surgical protocols and long‐term prognoses.

## 6. Conclusion

In summary, plough fractures represent a rare but distinct variant of TPAD. Hyperflexion and hyperextension injury mechanisms play pivotal roles in their pathogenesis. Diagnosis primarily relies on CT imaging. When clinical resources permit, upper cervical MRI provides critical information for this evaluation and is recommended to guide treatment. For patients with intact transverse ligaments, rigid external immobilization or internal fixation may be indicated based on fracture displacement and reducibility. Conversely, posterior C1–C2 arthrodesis consistently yields favourable outcomes in cases with transverse ligament disruption.

## Funding

This study was supported by China RongTong Medical Healthcare Group Co. Ltd. (No. 20240709411) through financial support and sponsorship to Haisong Yang.

## Conflicts of Interest

The authors declare no conflicts of interest.

## Data Availability

The data that support the findings of this study are available from the corresponding author upon reasonable request.
